# Ganoderic Acid D Protects Human Amniotic Mesenchymal Stem Cells against Oxidative Stress-Induced Senescence through the PERK/NRF2 Signaling Pathway

**DOI:** 10.1155/2020/8291413

**Published:** 2020-07-27

**Authors:** Yan Xu, Huan Yuan, Yi Luo, Yu-Jie Zhao, Jian-Hui Xiao

**Affiliations:** ^1^Zunyi Municipal Key Laboratory of Medicinal Biotechnology, Affiliated Hospital of Zunyi Medical University, Huichuan District, Zunyi 563003, China; ^2^Center for Translational Medicine, Affiliated Hospital of Zunyi Medical University, Huichuan District, Zunyi 563003, China; ^3^Department of Laboratory Medicine, Affiliated Hospital of Zunyi Medical University, Huichuan District, Zunyi 563003, China

## Abstract

Aging is an important risk factor in the occurrence of many chronic diseases. Senescence and exhaustion of adult stem cells are considered as a hallmark of aging in organisms. In this study, a senescent human amniotic mesenchymal stem cell (hAMSC) model subjected to oxidative stress was established *in vitro* using hydrogen peroxide. We investigated the effects of ganoderic acid D (GA-D), a natural triterpenoid compound produced from *Ganoderma lucidum*, on hAMSC senescence. GA-D significantly inhibited *β*-galactosidase (a senescence-associated marker) formation, in a dose-dependent manner, with doses ranging from 0.1 *μ*M to 10 *μ*M, without inducing cytotoxic side-effects. Furthermore, GA-D markedly inhibited the generation of reactive oxygen species (ROS) and the expression of p21 and p16 proteins, relieved the cell cycle arrest, and enhanced telomerase activity in senescent hAMSCs. Furthermore, GA-D upregulated the expression of phosphorylated protein kinase R- (PKR-) like endoplasmic reticulum kinase (PERK), peroxidase III (PRDX3), and nuclear factor-erythroid 2-related factor (NRF2) and promoted intranuclear transfer of NRF2 in senescent cells. The PERK inhibitor GSK2656157 and/or the NRF2 inhibitor ML385 suppressed the PERK/NRF2 signaling, which was activated by GA-D. They induced a rebound for the generation of ROS and *β*-galactosidase-positive cells and attenuated the differentiation capacity. These findings suggest that GA-D retards hAMSC senescence through activation of the PERK/NRF2 signaling pathway and may be a promising candidate for the discovery of antiaging agents.

## 1. Introduction

Aging is a major risk factor for most chronic diseases, such as metabolic, cardiovascular, and neurodegenerative diseases, and cancer [1]. Various studies have demonstrated that aging accelerates the initiation and/or clonal dominance of mutant stem cells in aging tissues, such as the intestinal epithelium, the hematopoietic system, and the male germline, which shows a causal relationship between the aging-associated accumulation of stem cell mutations and failure of tissue maintenance and cancer suppression [2]. In this regard, the senescence and exhaustion of adult stem cells are considered as a hallmark of aging in organisms. Adult stem cells are responsible for rejuvenating tissues by various processes, such as tissue maintenance, repair, and regeneration, throughout the life span of the higher organisms. However, a decline in their functions can lead to tissue dysfunction, organismal aging, and age-related diseases [3, 4]. For instance, aged hematopoietic stem cells gradually lose regenerative capacity [5], impair homing and engraftment upon transplantation, accumulate signatures of widespread DNA damage [6], drive aging-associated immune remodeling [7], and lead to hematological pathologies [8]. Likewise, mesenchymal stem cells (MSCs) are both subject to and key mediators of organismal aging [9]. Previous studies have shown that MSCs decrease in number and proliferative capacity as the body ages [10], as observed in the premature aging diseases including Werner syndrome and Hutchinson-Gilford premature aging syndrome, as well as in the aging mouse models [11, 12]. Furthermore, aged MSCs showed spontaneous expression of embryonic factors and p53 point mutations in an age-related tumorigenesis model [13]. Interestingly, transplantation of mesodermal-derived stem cells into aging mice can prolong their life span [14], while senescent bone marrow MSCs cannot reach the injury site and lose their protection to the lungs due to weak activation, migration, and anti-immune function [15]. Hence, stem cell senescence has received increased attention in recent years in the field of antiaging and regenerative medicine.

*Ganoderma lucidum* (Leyss. Ex. Fr) Karst, a medicinal mushroom, also referred to as “Lingzhi,” has been widely used as a famous traditional Chinese medicine to promote health and longevity for thousands of years in China and other Oriental countries. Furthermore, recent pharmacological studies have demonstrated that the extracts of *G. lucidum*, such as aqueous extracts and ethanol extracts, induce antiaging effects by increasing the mitochondrial antioxidant activity, scavenging free radicals, and reducing the generation of reactive oxygen species (ROS) [16–18]. Ethanol extracts of *G. lucidum* have delayed the progression of age-related Alzheimer's disease by regulating the methylation level of DNA [18]. However, the antiaging bioactive ingredients in *G. lucidum* have not been elucidated. One study showed that several ergosterol derivatives, ganodermasides A, B, C, and D isolated from the methanol extract of spores of *G. lucidum*, prolonged the replicative life span of yeast by targeting an age-related gene *UTH1* [16]. More than 200 distinct chemical entities with various pharmacological actions, such as antioxidation, antitumor, and antiradiation effects, have been isolated from *G. lucidum* [19, 20]. However, the number of confirmed antiaging ingredients is lower than the large number of known compounds that have been isolated from *G. lucidum*. The dysfunction of antioxidant enzymes accelerates the aging process, as they are the first line of defense for protecting biological macromolecules against oxidative stress. Although there are no reports on the antiaging activity of *G. lucidum* triterpenes, their antioxidant properties suggest that they may have a potential effect on the extension of life span.

Senescent cells accumulate in various aging tissues and at pathogenic sites in many chronic diseases. Notably, targeting cellular senescence is regarded as a promising approach for the delay, prevention, or alleviation of multiple age and cellular senescence-associated conditions and the fundamental aging processes [21]. The free radical theory of aging postulates that the production of intracellular ROS is the major determinant of life span. What is the link between ROS and senescence? Excessive accumulation of ROS induces oxidative damage in cells. A previous study showed that oxidative damage contributed to replicative senescence [22]. Oxidative stress triggers DNA damage, resulting in the dysregulation of cell homeostasis and aging phenotypic characteristics, eventually leading to the acceleration of the cellular senescence [23]. Additionally, H_2_O_2_-induced oxidative damage could cause the cellular and molecular changes in senescent cells. For example, both p21 and p16 proteins are elevated during the induction of premature senescence, thereby causing cell cycle arrest and loss of self-replication [22]. Thus, aging and age-related diseases are regulated by intracellular free radicals, and generation of ROS remains one of the most widely accepted causes of aging [24]. Therefore, it is an effective strategy to reduce the excessive accumulation of ROS to slow down the senescence of MSCs.

Based on the free radical theory, we developed a H_2_O_2_-induced stem cell senescent model using human amniotic MSCs (hAMSCs) with high expression of *β*-galactosidase, a senescence-associated marker. Dozens of natural compounds isolated from *G. lucidum* were screened using the H_2_O_2_-induced hAMSC senescent model. Among these compounds, ganoderic acid D (GA-D), a triterpenoid compound, dramatically alleviated stem cell senescence. Therefore, in the present study, we have reported the effect of GA-D on oxidative stress-induced stem cell senescence as well as the underlying mechanism of delayed senescence in hAMSCs.

## 2. Materials and Methods

### 2.1. Source and Identification of GA-D Compound

**Figure 1:**
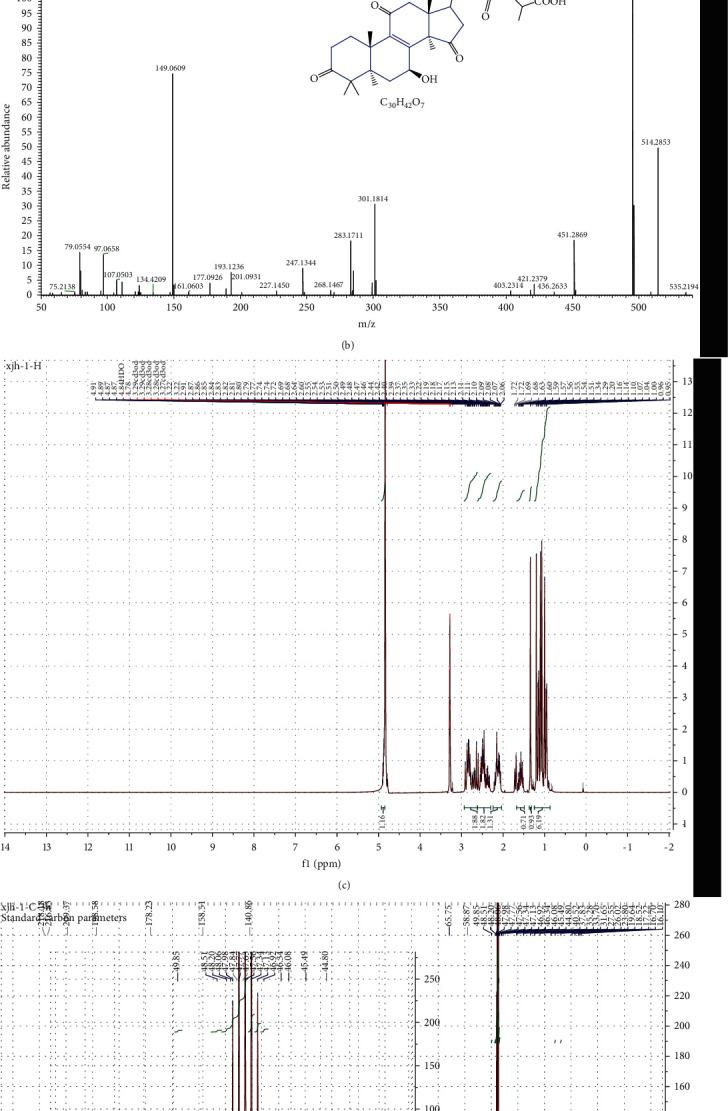
Characterization of the GA-D. (a) HPLC analysis; (b) molecular formula and mass spectrometry data. ESI-MS at *m*/*z* 513.3 [M-H]^−1^. (c, d) The data of ^13^C- and ^1^H-NMR spectra.

The GA-D compound was purchased from Baoji Chenguang Biotechnology Company, Baoji, China. The properties of GA-D, such as purity, molecular weight, and chemical structure, were identified using high-performance liquid chromatography (HPLC), liquid chromatography-mass spectrometry (LC-MS), and nuclear magnetic resonance (NMR), respectively. The HPLC-grade purity was observed to be above 98% by HPLC analysis (Figure 1(a)), and the molecular formula was determined to be C_30_H_42_O_7_ through ESI-MS (negative) at a mass to charge ratio (*m*/*z*) of 513.3 [M-H]^−1^ (Figure 1(b)). Consistent with its molecular formula, 30 carbon resonances were observed in the ^13^C-NMR spectrum, and the data of ^13^C- and ^1^H-NMR spectra (Figures 1(c) and 1(d)) were consistent with the compound ganoderic acid D identified by Kikuchi et al. [25]. Consequently, the compound was confirmed to be ganoderic acid D.

### 2.2. Cell Isolation, Culture, and Identification

As per previously described methods [26, 27], hAMSCs were isolated from placental amnion tissue that was collected from normal pregnant women after gaining informed consent using collagenase type II (Solarbio, Beijing, China) and deoxyribonuclease I (Solarbio, Beijing, China) enzymes. The hAMSCs were cultured in Dulbecco's modified Eagle medium low glucose (LG-DMEM) (Gibco, New York, USA) supplemented with 10% fetal bovine serum (FBS) (Gibco, New York, USA), 1% nonessential amino acids (Gibco, New York, USA), 10 ng/mL basic fibroblast growth factor (bFGF) (Peprotech, NJ, USA), and 1% *L*-alanyl-*L*-glutamine dipeptide (*L*-GlutaMAX) (Gibco, New York, USA) in a humid atmosphere of 5% CO_2_ at 37°C. Culture medium was replaced by fresh medium every three days. When the cells reached 80% confluency, the harvested cells were passaged. Cells that belonged to passage 2 (P2) were used for further analysis in the study. Subsequently, these hAMSCs were analyzed using flow cytometry and the immunocytochemical staining method according to the protocols described in previous studies [26, 27]. This research was conducted in accordance with the Declaration of Helsinki and the guidelines of the Ethical Committee of the Affiliated Hospital of Zunyi Medical University (Zunyi China).

### 2.3. Cell Treatment

When P3 hAMSCs were grown to a confluency of approximately 50%, they were treated with H_2_O_2_ at different final concentrations (100 *μ*M, 200 *μ*M, and 400 *μ*M) for 2 h. Subsequently, the cells were washed with Dulbecco's phosphate-buffered saline (D-PBS) to remove residual H_2_O_2_ and replaced with fresh LG-DMEM/F12 complete medium. The aging-related indicators were tested after the cells were cultured up to the specified time point. Different final concentrations (0.001 *μ*M, 0.01 *μ*M, 0.1 *μ*M, 1 *μ*M, 10 *μ*M, and 100 *μ*M) of GA-D were used to pretreat hAMSCs for 6 h prior to the addition of H_2_O_2_. The protein kinase R- (PKR-) like endoplasmic reticulum kinase (PERK) inhibitor GSK2656157 (MCE, Shanghai, China) and nuclear factor-erythroid 2-related factor (NRF2) inhibitor ML385 (MCE, Shanghai, China) were added 1 h prior to the addition of GA-D. GA-D, GSK2656157, and ML385 were dissolved in dimethylsulfoxide (DMSO) (Solarbio, Beijing, China) at a concentration of 10 mM and diluted to the corresponding concentration with D-PBS prior to use.

### 2.4. Flow Cytometry Analysis

For the phenotypic characterization of hAMSCs, a BD Stemflow™ Human MSC analysis kit (Cat. No. 562245, BD Biosciences, San Diego, CA, USA) was used in the study. P3 hAMSCs in the logarithmic growth phase were harvested and labeled with different antibodies for human MSC-specific markers (CD73, CD90, and CD105) for flow cytometry analysis. Briefly, P3 hAMSCs were collected and washed twice with D-PBS containing 0.1% BSA, adjusted to a density of 1 × 10^6^ cells/mL, and then incubated with the corresponding antibody for 1 h in the dark. After washing again with D-PBS containing 0.1% BSA, the cell suspension was centrifuged at 1000 rpm for 5 min and the supernatant was discarded. Finally, the labeled cells were analyzed by flow cytometry (BD, Franklin Lakes, NJ, USA) using the CellQuest software after fixation with 1% paraformaldehyde.

### 2.5. Senescence-Associated *β*-Galactosidase Staining

Cell senescence was detected using a *β*-galactosidase staining kit (Beyotime, Shanghai, China). The *β*-galactosidase dyeing working solution was prepared according to the manufacturer's instructions. The hAMSCs were fixed for 15 min after washing once with D-PBS. Then, the cells were washed thrice with PBS and stained with *β*-galactosidase stain working solution for 4 h at 37°C.

### 2.6. Telomerase Activity Assay

Telomerase activity in cell extracts was measured using a telomerase detection kit (Elabscience, Wuhan, China). The cells were digested and collected using trypsin. Thereafter, the cell pellet was mixed with an appropriate amount of D-PBS, and the cell membrane was disrupted by freezing and thawing the cells repeatedly for protein extraction. Following this, the supernatant was collected and stored at -20°C. The subsequent steps were accomplished according to the manufacturer's instructions.

### 2.7. Cell Viability Assay

Cell viability was determined using the MTT assay. The cells were mixed with the MTT solution and incubated for 4 h in an incubator. Thereafter, the solution was aspirated, and DMSO was added to dissolve the precipitate. Subsequently, the absorbance was measured at 450 nm. Relative inhibition rate can be calculated using the following formula: relative proliferation rate (X group) = (1 − (X group average OD − blank control group average OD)/(control group average OD − blank control group average OD))∗100%, wherein the X group, the control group, and the blank control group are the GA-D-treated group, the untreated group, and the culture medium group without cells and GA-D, respectively.

### 2.8. Intracellular Reactive Oxygen Species (ROS) Assay

An ROS detection kit (Beyotime, Shanghai, China) was used to measure the content of intracellular ROS. Cells were treated with 2,7-dichlorodihydrofluorescein diacetate (DCFH-DA) dilution solution after washing once with D-PBS and incubated for 30 min. The level of intracellular ROS was observed using the Nikon Eclipse Ti-S inverted fluorescence microscope (Nikon, Tokyo, Japan).

### 2.9. Osteogenic and Chondrogenic Differentiation of hAMSCs In Vitro

Approximately 2 × 10^4^ cells were seeded in 12-well plates in LG-DMEM culture medium. After incubation for 24 h, the cells were treated with GA-D and replaced with osteoblast-conditioned medium (HG-DMEM (Gibco, New York, USA) with 10% FBS, 100 nmol/L dexamethasone (Sigma, SL, USA), 50 mg/L vitamin C (Solarbio, Beijing, China), and 10 mmol/L *β*-glycerophosphate (Solarbio, Beijing, China)) and chondrocyte-conditioned medium (HG-DMEM with 10 ng/L transforming growth factor- (TGF-) *β*3 (Peprotech, NJ, USA), 1 × 10^−7^ mol/L dexamethasone, and 50 mg/L vitamin C) for 7 days, as described in previous reports [26, 28]. Osteogenic differentiation was preliminarily evaluated by alkaline phosphatase staining, and chondrogenesis was assessed by toluidine blue staining.

### 2.10. Cell Cycle Analysis

The cell cycle was detected using a DNA content quantitation assay kit (Solarbio, Beijing, China). Cells were collected and digested using trypsin and washed with D-PBS. The cells were fixed by the addition of prechilled 70% ethanol followed by overnight incubation. Thereafter, the cells were resuspended in RNase A and incubated at 37°C for 30 min. Subsequently, propidium iodide staining solution was added and incubated at 4°C for 30 min. Cell cycle distribution was measured using flow cytometry.

### 2.11. Quantitative Real-Time PCR Analysis

Total RNA from hAMSCs was extracted using RNAiso Plus (Takara, Dalian, China), and 1-2 *μ*g of the total RNA was used for cDNA synthesis with the PrimeScript™ RT reagent kit (Takara, Dalian, China). Real-time PCR was performed and monitored using the SYBR® Premix Ex Taq II (Takara, Dalian, China) quantitative real-time PCR system, and *β*-actin was used as the loading control. The threshold cycle (Ct) value was used to calculate the relative expression of genes. The primers for all genes tested are listed in Table 1.

### 2.12. Western Blotting

Total protein of hAMSCs was extracted using the radioimmunoprecipitation assay (RIPA) lysis buffer (Solarbio, Beijing, China), and nuclear protein was extracted using a nuclear protein extraction kit (Solarbio, Beijing, China). Proteins were detected through SDS-PAGE and subsequently electrotransferred to polyvinylidene fluoride membranes. The antibodies used were as follows: anti-p21 antibody (Abcam, ab80633), anti-p16 antibody (HuaBio, Hangzhou, China), anti-Nrf2 antibody (Abcam, ab62352), anti-PERK antibody (CST, 3192S), anti-p-PERK antibody (CST, 3179S), and anti-peroxidase III (PRDX3) antibody (Abcam, ab128953). Then, membranes were incubated with horseradish peroxidase conjugated-secondary antibody (ProteinTech, SA00001-2) for 2 h at room temperature. Finally, the membranes were developed using enhanced chemiluminescence (ECL) hypersensitive luminescent solution (Beyotime, Shanghai, China). The intensity of the protein bands was analyzed using ImageJ software.

### 2.13. Statistical Analysis

All experiments were performed at least three times, and the data were expressed as the mean ± standard error of the mean (SEM). The GraphPad Prism software was used to conduct Student's *t*-test and analyze the data. *p* < 0.05 was considered statistically significant.

## 3. Results

### 3.1. Identification of hAMSCs and Establishment of a Senescent hAMSC Model

Similar to our previous studies [28, 29], the surface molecules of MSCs were highly expressed in hAMSCs, including CD105 (88.10%), CD73 (99.84%), and CD90 (98.48%). However, the expression of cell surface molecules of hematopoietic stem cells (0.12%), including CD34, CD11b, CD19, CD45, and HLD-AR, was not observed. Additionally, hAMSCs strongly expressed vimentin (a marker protein of MSCs) but did not express cytokeratin 19 (a marker protein of epithelial cells) (Supplementary Figure 1). These results indicate that hAMSCs conform to MSC accreditation, as recommended by the International Society for Cellular Therapy [30].

**Figure 2:**
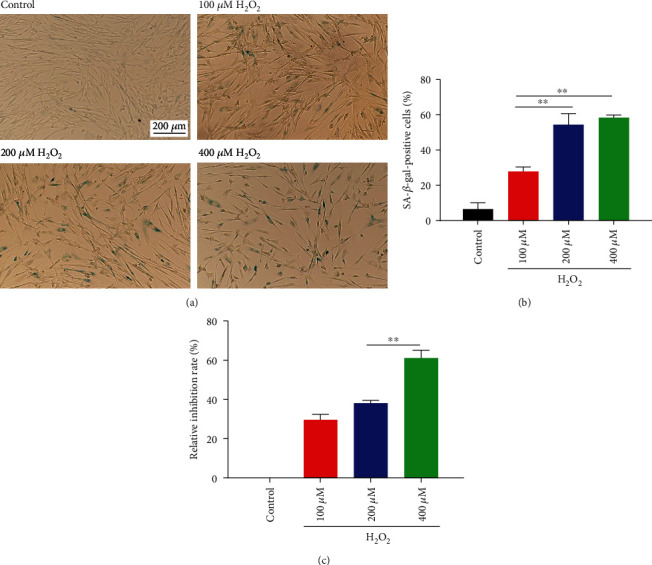
H_2_O_2_ treatment induced the premature cellular senescence of hAMSCs. (a) Cell density and SA-*β*-gal-positive cells. Scale bar = 200 *μ*m. (b) Statistics of SA-*β*-gal-positive cell rates. (c) Effects of different H_2_O_2_ concentrations on hAMSC viability. Data are presented as mean ± SEM, *n* = 3, ^∗∗^*p* < 0.01.

To verify the effects of oxidative stress on hAMSCs, H_2_O_2_ was used to treat them. The formation of the senescence marker *β*-galactosidase and cell viability were detected by *β*-galactosidase staining and MTT assay, respectively. As shown in Figure 2(a), H_2_O_2_ induced significantly premature cellular senescence of hAMSCs, as indicated by decreased cell density and increased rate of SA-*β*-gal-positive cells, as compared to the control group. The rate of SA-*β*-gal-positive cells was increased to 54.33% ± 5.15% and 58.33% ± 1.24% after treatment with 200 *μ*M H_2_O_2_ and 400 *μ*M H_2_O_2_, respectively. Though no significant difference was observed between the two groups (Figure 2(b)), the MTT assay showed that 400 *μ*M H_2_O_2_ was more toxic to cells than 200 *μ*M H_2_O_2_ (Figure 2(c)).

### 3.2. GA-D Inhibits the Generation of *β*-Galactosidase

**Figure 3:**
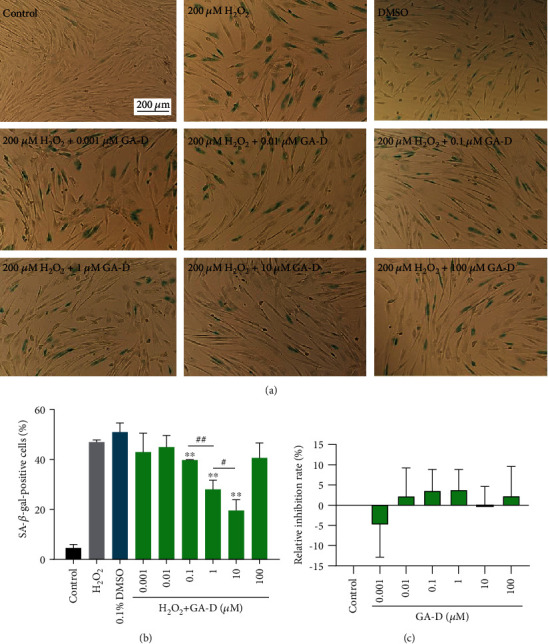
GA-D significantly reduced the generation of *β*-galactosidase. (a) The effect of different concentrations of GA-D on H_2_O_2_-induced premature senescence in hAMSCs by *β*-galactosidase staining; 0.1% DMSO group was used as a solvent control. Scale bar = 200 *μ*m. (b) Statistical analysis of SA-*β*-gal-positive cell rates. (c) Effect of different concentrations of GA-D on hAMSC viability through MTT assay. GA-D: ganoderic acid D. Data are presented as mean ± SEM, *n* = 3, ^#^*p* < 0.05, ^##^*p* < 0.01, ^∗∗^*p* < 0.01 vs. H_2_O_2_ group.

To investigate the effects of GA-D on senescence induced by oxidative stress in hAMSCs, GA-D was used to pretreat hAMSCs prior to H_2_O_2_ treatment. The generation of *β*-galactosidase was suppressed in a dose-dependent manner when 0.1 *μ*M, 1.0 *μ*M, and 10.0 *μ*M GA-D were employed (Figure 3(a)), but 100 *μ*M GA-D had no significant effect against the senescent cells (Figures 3(a) and 3(b)). The data showed that the proportion of the SA-*β*-gal-positive cell rate was reduced from 46.92% ± 0.74% to 39.7% ± 0.17%, 28.0% ± 2.9%, and 19.58% ± 3.5%, at the respective concentrations (Figure 3(b)). The inhibitory rate against the generation of *β*-galactosidase of 10 *μ*M GA-D reached 58.3%. Moreover, GA-D at various concentrations ranging from 0.001 *μ*M to 100.0 *μ*M was nontoxic to normal hAMSCs (Figure 3(c)). Therefore, these results suggest that 10 *μ*M GA-D exerts positive effects on inhibition of H_2_O_2_-induced cellular senescence in hAMSCs.

### 3.3. GA-D Prevents H_2_O_2_-Induced Premature Senescence of hAMSCs

**Figure 4:**
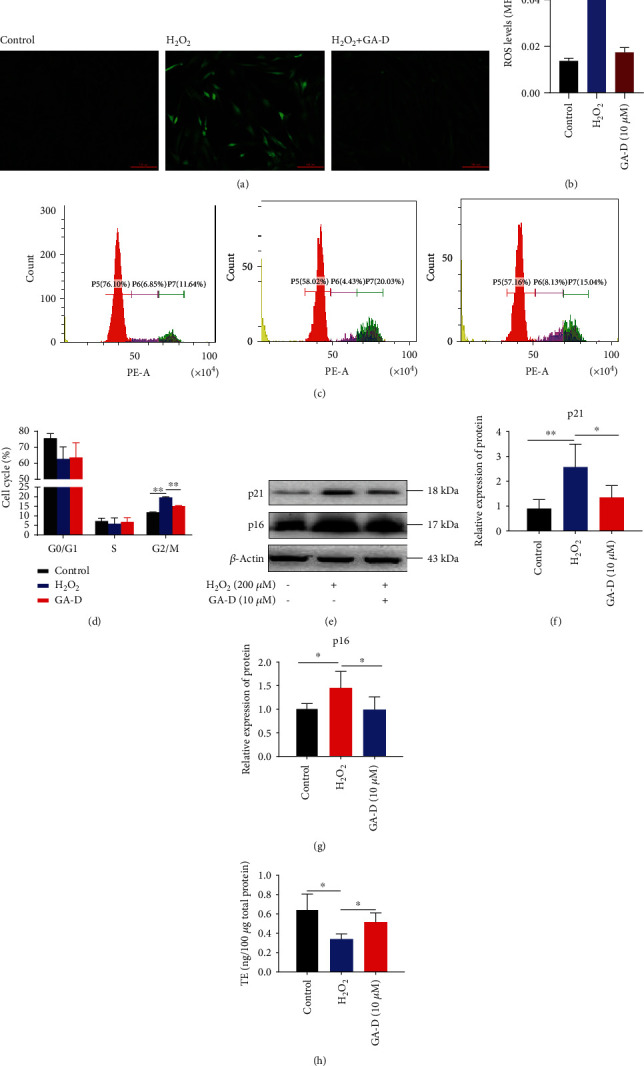
Pretreatment with GA-D could significantly prevent H_2_O_2_-induced premature senescence of hAMSCs. (a, b) Intracellular ROS levels were detected after pretreatment of GA-D. Scale bar = 200 *μ*m. (c, d) The influence of GA-D on H_2_O_2_-induced cell cycle distribution. (e–g) Relative expression of p21 and p16^INK4a^. (h) Production of telomerase. GA-D: ganoderic acid D. Data are presented as mean ± SEM, *n* = 3, ^∗^*p* < 0.05, ^∗∗^*p* < 0.01.

To confirm the preventive effect of GA-D on H_2_O_2_-induced senescence of hAMSCs, we further detected the production of intracellular ROS, cell cycle arrest, telomerase activity, and other senescence indicators. The production of intracellular ROS (Figures 4(a) and 4(b)) was obviously inhibited by GA-D at 10 *μ*M. Additionally, H_2_O_2_ caused cell cycle arrest in the G2/M phase from 11.84% ± 0.16% to 19.64% ± 0.28%, but GA-D partially rescued this change of cell cycle from 19.64% ± 0.28% to 15.11% ± 0.20% in the G2/M phase (Figures 4(c) and 4(d)). GA-D also significantly downregulated the relative expression of p21 and p16^INK4a^ at the translational level in senescent cells (Figures 4(e)–4(g)). Interestingly, the production of telomerase was also increased with the addition of GA-D (Figure 4(h)). These results further indicated that GA-D could prevent H_2_O_2_-induced senescence of hAMSCs.

### 3.4. GA-D Prevents Senescence of hAMSCs by Activating the PERK/NRF2 Signaling Pathway

**Figure 5:**
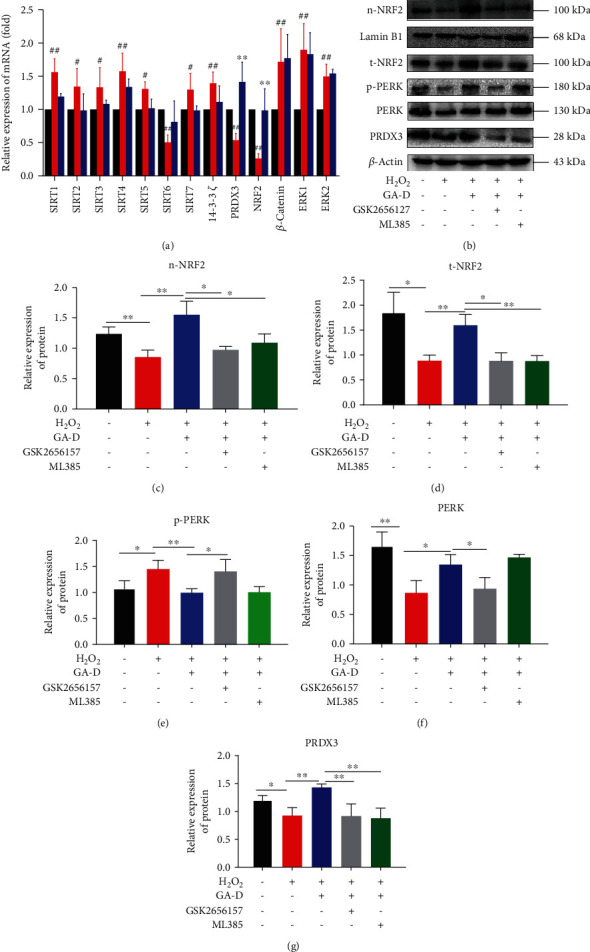
Antisenescence effect of GA-D on hAMSCs through the regulation of the PERK/NRF2 signaling pathway. (a) Changes in senescence-related genes after GA-D pretreatment. (b) Changes in n-NRF2, t-NRF2, p-PERK, PERK, and PRDX3 expression in hAMSCs treated with different treatments. (c, d) Relative expression levels of n-NRF2 and t-NRF2. (e, f) Relative expression levels of p-PERK and PERK. (g) Relative expression level of PRDX3. Normal control group (black); H_2_O_2_-induced model group (red); GA-D group (blue); GA-D plus GSK2656157 group (gray); GA-D plus ML385 group (green). GA-D: ganoderic acid D; n-NRF2: nuclear NRF2 protein; t-NRF2: total NRF2 protein; p-PERK: phosphorylated PERK protein. Data are presented as mean ± SEM, *n* = 3, ^∗^*p* < 0.05, ^∗∗^*p* < 0.01.

In the present study, we analyzed the transcriptional levels of some key genes that played an important role in the senescence process through qRT-PCR. The transcriptional levels of all genes analyzed were significantly altered in senescent hAMSCs. However, only NRF2 and PRDX3 were dramatically upregulated after the treatment with GA-D (Figure 5(a)), and this change was also observed at the translational level (Figure 5(b)). Furthermore, it was observed that GA-D promoted intranuclear transfer of NRF2 in senescent hAMSCs (Figure 5(c)). Generally, PERK delays cell cycle arrest by activating NRF2 [31], thereby indicating that the PERK/NRF2 signaling pathway might involve cellular senescence. Therefore, it is questionable whether there is a link between PERK and NRF2 in the antisenescence process of GA-D. We examined the protein expression levels of PERK in this study. Consistent with our prediction, the protein expression levels of phosphorylated PERK (p-PERK) were downregulated in senescent hAMSCs; however, they were upregulated after GA-D pretreatment (Figure 5(e)). Additionally, we found that GSK2656157 not only inhibited the expression of p-PERK (Figure 5(e)) but also reduced the expression of NRF2 in the nucleus (n-NRF2) and total NRF2 (t-NRF2) proteins (Figures 5(c) and 5(d)). However, only the expression of NRF2 (t-NRF2 and n-NRF2) could be suppressed in the presence of ML385 (Figures 5(c) and 5(d)), and there was no evident effect on p-PERK (Figure 5(e)). None of the treatments affected the expression of PERK protein, except for the H_2_O_2_-induced model group (Figure 5(f)). However, the expression of PRDX3 was inhibited when GSK2656157 or ML385 was employed (Figure 5(g)). These results suggest that GA-D might activate p-PERK signaling to promote intranuclear transfer of NRF2 and ultimately prevent the senescence of hAMSCs.

### 3.5. Antisenescence Effect of GA-D Is Attenuated after the Inhibition of the PERK/NRF2 Signals

**Figure 6:**
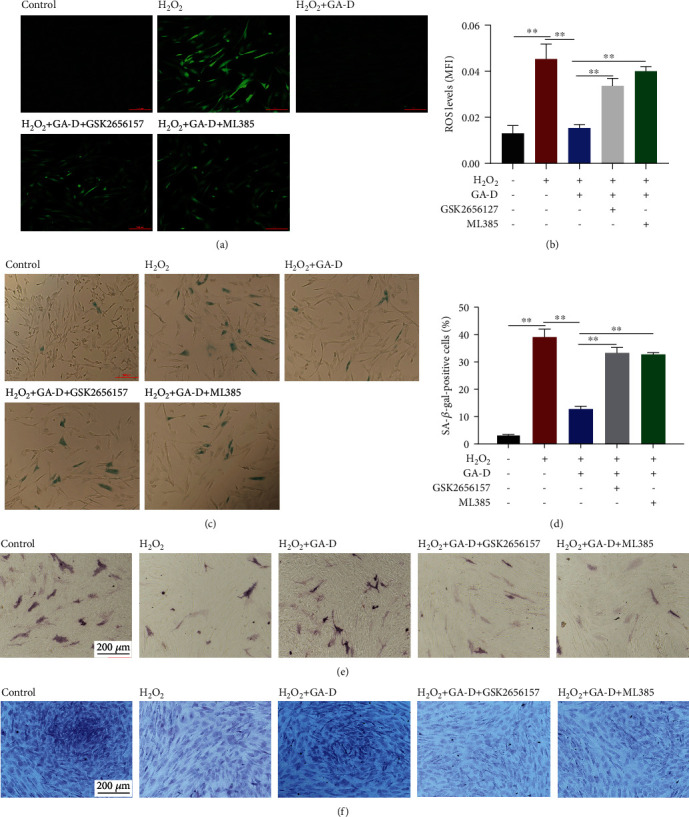
Antisenescence of GA-D was attenuated after inhibition of the PERK/NRF2 signaling pathway. (a, b) Intracellular ROS levels after inhibition of PERK and NRF2 expression. Scale bar = 200 *μ*m. (c, d) Changes of *β*-galactosidase expression in hAMSCs after inhibition of PERK and NRF2 expression. Scale bar = 200 *μ*m. Data are presented as mean ± SEM, *n* = 3, ^∗∗^*p* < 0.01. (e) The ability of hAMSCs to differentiate into osteoblasts was analyzed by alkaline phosphatase staining. Scale bar = 200 *μ*m. (f) The capability of hAMSCs to differentiate into chondrocytes was detected by toluidine blue staining. GA-D: ganoderic acid D. Scale bar = 200 *μ*m.

To verify whether GA-D delays the oxidative stress-induced senescence of hAMSCs through the activation of the PERK/NRF2 signals, the production of ROS and *β*-galactosidase was detected in hAMSCs in the presence of GSK2656157 and/or ML385. As shown in Figures 6(a)–6(d), the ROS level significantly increased after the blockage of PERK/NRF2 signals (Figures 6(a) and 6(b)), along with an increase in the generation of *β*-galactosidase (Figures 6(c) and 6(d)). As previously described, the senescence of MSCs led to a decrease in the ability to differentiate into osteoblasts and chondrocytes [32]. Therefore, the osteoblastic and chondrocytic differentiation capabilities of hAMSCs were preliminarily detected through alkaline phosphatase staining and toluidine blue staining, respectively. The expression of alkaline phosphatase (Figure 6(e)) and glycosaminoglycan (Figure 6(f)) was significantly decreased in senescent hAMSCs compared to the normal group, indicating that the differentiation ability of senescent hAMSCs was markedly attenuated, and this change could be restored by GA-D in senescent hAMSCs. However, the antisenescent effect of GA-D was reversed after the addition of GSK2656257 and ML385 (Figures 6(e) and 6(f)). Therefore, these results suggest that the antisenescence effect of GA-D activates PERK/NRF2 signals in senescent hAMSCs.

## 4. Discussion

Stem cell aging is regarded as an important driver of organism aging. Adult stem cells are essential for maintaining tissue and organ homeostasis through repair and regeneration during life. For instance, adipose-derived mesenchymal stem cells derived from old horses exhibited a typical senescence phenotype and limited regenerative capacity [33]. Aging-associated phenotypes can be reversed *in vivo* through the induction of stem cell rejuvenation [34]. Supporting this notion, delayed senescence of stem cells might be a promising novel strategy for ameliorating organismal aging and treating aging-related diseases. *Ganoderma lucidum* is a well-known traditional Chinese tonic that promotes health and longevity in East Asian countries. Recent studies have demonstrated that the extracts and polysaccharides of *Ganoderma lucidum* have distinct roles in delaying aging and the treatment of aging-related diseases through a multitargeted mechanism [16–18]. Polysaccharides and triterpenoids are the two major bioactive components of *Ganoderma lucidum*. Furthermore, *Ganoderma lucidum* is regarded as a cellular factory that produces a diverse set of bioactive triterpenoid compounds. So far, more than 210 triterpenoid compounds with different chemical structures have been identified [19, 20]. To our knowledge, however, the antiaging bioactivity of *Ganoderma lucidum*-derived triterpenoid compounds remains unknown [35]. Among all natural small-molecule compounds produced from *Ganoderma lucidum*, only four novel ergosterol derivatives, ganodermasides A–D, were shown to prolong the replicative life span of yeast through the regulation of the aging-related gene *UTH1* [16]. In the present study, the protective effect of GA-D, a *Ganoderma lucidum*-derived triterpenoid compound against oxidative stress-induced stem cell senescence, was reported for the first time. Furthermore, GA-D inhibited the generation of ROS and senescence-associated markers, such as *β*-galactosidase, p21, and p16^INK4a^, and enhanced telomerase activity through the activation of the PERK/NRF2 signaling pathway.

Based on recently reported data [35, 36], antiaging activity was observed for more than 200 natural small-molecule compounds that were produced in plants and fungi. Among these natural compounds, saponin compounds with a tetracyclic triterpenoid skeleton, such as ginsenosides Rg1, Rb1, and Rg3 from *Panax ginseng* CA Mey and cycloastragenol from *Astragalus propinquus* Schischkin, showed potent antiaging effects as did oleanolic acid from *Fructus ligustri* Lucidi, a triterpenoid with a pentacyclic structure. To date, ganoderic acid A, a *Ganoderma lucidum*-derived triterpenoid compound, was found to enhance antioxidant enzyme activity, inhibit ROS production, and increase the mitochondrial membrane potential [37]. The increased ROS and loss of the mitochondrial membrane potential were important inductive factors of cellular senescence [38], implying that *Ganoderma lucidum*-derived triterpenoid compounds may have antiaging potential by inhibiting oxidative stress. Consistent with these findings, GA-D also showed potent antiaging activity in hAMSCs by inhibiting oxidative stress. Therefore, natural triterpenoid compounds and their derivatives containing triterpenoid skeletons might exert strong antiaging potentials. Our findings suggest that GA-D is the first antiaging tetracyclic triterpenoid compound. However, further investigation *in vivo* is needed to confirm its antiaging activity.

Oxidative stress is an important factor that causes cellular senescence and body aging, and high concentrations of ROS trigger DNA damage and lead to cellular senescence through the direct or indirect regulation of aging-related signaling pathways [39]. As described in previous studies [16–18], *Ganoderma lucidum* could exert antiaging effects by regulating oxidative stress. In the present study, a senescent hAMSC cell model subjected to oxidative stress was established *in vitro* using H_2_O_2_. While GA-D can evidently decrease the ROS, *β*-galactosidase, p21, and p16^INK4a^ levels and increase the content of telomerase in senescent hAMSCs, the underlying mechanism of the antisenescence effect of GA-D remains unclear. The regulatory mechanisms of stem cell senescence are extremely complex. Previous studies have shown that various signals, such as Keap1/NRF2 [40], Wnt/*β*-catenin [41], and ERK1/2 [42], are closely related to stem cell senescence. Sirtuins, which are metabolic sensors, are recognized as a link between the metabolic signaling and senescence. The life span-extending effects of dietary restriction involved the activation of sirtuins, and members of the sirtuin family, including SIRT1 [43], SIRT3 [44], and SIRT6 [45], could delay the senescence of stem cells by regulating oxidative stress. 14-3-3*ζ*, as a possible binding site of GA-D in the cell [46], played an important role in imposing resistance to ROS stimulation [47]. Additionally, a decrease in PRDX3 could cause oxidative stress and mitochondrial dysfunction to induce senescence in trophoblast cells [48]. A recent study reported that the ethanol extracts of *Ganoderma lucidum* could reduce the production of ROS by increasing the expression and phosphorylation of NRF2 to induce the upregulation of heme oxygenase 1 (HO-1) [49]. Based on these data, we analyzed the changes in some key genes of these signals in senescent hAMSCs induced by H_2_O_2_ in the presence of GA-D. All genes that were analyzed showed a dramatic change at the transcriptional level in senescent hAMSCs compared to the control group, indicating that the aging model was successfully established. However, only the relative expression levels of NRF2 and PRDX3 were upregulated after pretreatment with GA-D, thereby indicating that the function of GA-D against oxidative stress-induced hAMSC senescence may be associated with NRF2 and PRDX3 signals. As described previously [50], the expression of NRF2 was considerably reduced in senescent cells, leading to an alteration in the redox balance. Meanwhile, the senescence of cells accelerated when NRF2 was absent [51]. Moreover, extracts of *Ganoderma lucidum* inhibited oxidative stress by enhancing the expression of NRF2 in the ovarian cancer cell line OVCAR-3 [52]. Additionally, NRF2 could reduce oxidative stress by regulating the expression of PRDX3 [53]. Therefore, consistent with the results of previous studies, the antisenescence effect of GA-D implicates antioxidant NRF2 signaling.

PERK is a type I transmembrane protein kinase receptor that is activated by ER stress, and NRF2 is a central transcriptional activator for cytoprotective target genes. The PERK/NRF2 antioxidant signaling pathway plays an important role in oxidative stress and ER stress [54]. Oxidative and ER stresses are triggered by the accumulation of ROS in cells. Subsequently, PERK and NRF2 are activated to enhance the expression of antioxidant enzymes and detoxification enzymes that restore redox homeostasis [55]. Moreover, activation of PERK also enhances cell resistance to oxidative stress by promoting nuclear transfer of NRF2 [56]. Therefore, we speculated that the PERK signal might be activated in senescent hAMSCs after pretreatment with GA-D. Similar to the expression of NRF2 at the translational level, the subsequent detection showed that GA-D gave rise to the upregulation of PERK, especially at the phosphorylation level of PERK. Furthermore, when the specific inhibitors, including GSK2656157 for PERK signaling and ML385 for NRF2 signaling, were employed, the corresponding signaling not only was blocked but also showed mutual blocking (Figures 5(c)–5(f)). At the same time, the antisenescence properties of GA-D were reversed in senescent hAMSCs (Figure 6). Therefore, two inhibitors could individually result in an increase in ROS levels and *β*-galactosidase and the diminished differentiation potential of hAMSCs in the GA-D treatment group. Consistent with the results of a previous study [53], our results showed that GA-D activated the expression of PERK, thereby promoting the intranuclear transfer of NRF2 to enhance the expression of PRDX3. Additionally, the activity of NRF2 was regulated by PERK, which activated NRF2 to reduce cell cycle arrest caused by oxidative stress [31]. Therefore, these results indicated that GA-D delayed the senescence of hAMSCs by activating the PERK/NRF2 signaling pathway.

**Figure 7:**
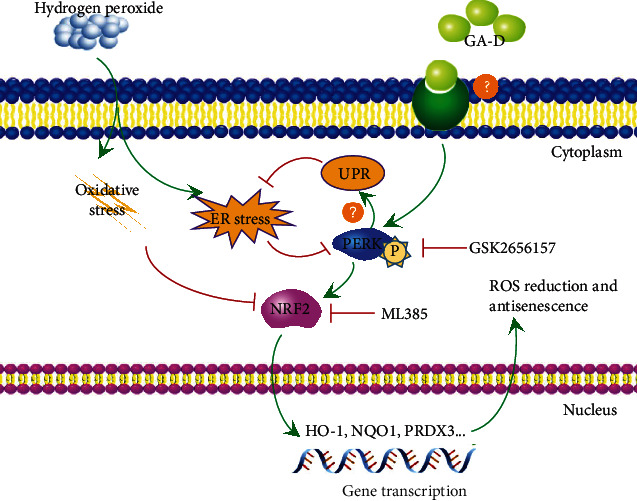
Schematic diagram showing the proposed antisenescence mechanism of GA-D in hAMSCs. According to this model, GA-D activating PERK results in intranuclear transfer of NRF2, which then upregulates the expression of PRDX3 and inhibits ROS. Additionally, the activation of PERK also inhibits ERS by triggering UPR, overall resulting in inhibition of senescence.

As already described, the accumulation of ROS triggers the ER stress, which leads to a disturbance in the function of ER and accumulation of misfolded proteins [57]. The unfolded protein response (UPR), a cell protection program, is initiated to reduce the production of misfolded proteins and to finally extend the life span [58]. PERK is a key protein that mediates the occurrence of UPR. PERK autophosphorylation activates the eukaryotic promoter, eukaryotic initiation factor 2*α* (eIF2*α*), which reduces the initiation of translation of most proteins in cells, thereby reducing the ER load. In the present study, it appears that H_2_O_2_ induces oxidative stress and ER stress in hAMSCs and finally induces cell senescence by increasing the accumulation of intracellular ROS and misfolded proteins. After GA-D pretreatment, the phosphorylation level of PERK was upregulated (Figure 5(e)), which might have mediated the occurrence of UPR to reduce ER stress. Meanwhile, p-PERK promoted the expression of NRF2 and nuclear transfer (Figures 5(c) and 5(d)) and finally triggered an increase in the transcription of downstream genes of NRF2, including *PRDX3*, *HO-1*, and quinine oxidoreductase 1 (*NQO1*) [53]. Consequently, hAMSC senescence was mitigated by decreased accumulation of ROS. This effect can be reversed by using specific inhibitors of PERK and/or NRF2; however, further investigation is required. Moreover, the mechanism underlying the delay in senescence of hAMSCs by GA-D remains unclear. The results of ligand-protein inverse-docking (INVDOCK) analysis suggested that GA-D could bind six isoforms of the 14-3-3 protein family, annexin A5, and aminopeptidase B [46]. Therefore, we speculate that GA-D may bind to one of these receptors for its functions; however, this needs to be further explored. Based on the above-mentioned analysis, a schematic diagram presenting the possible antisenescence mechanism of GA-D in hAMSCs can be speculated, as shown in Figure 7. To summarize, we report for the first time that natural triterpenoid compounds attenuate stem cell senescence *in vitro* through the activation of the PERK/NRF2 signals, and NRF2 is traceable as a key gene in the antisenescence effect of GA-D. These findings offer a new candidate agent for the prevention of aging-related diseases. However, due to the complexity of the signal regulation pathway, further studies on the signaling regulatory network of GA-D in senescent hAMSCs as well as its therapeutic effect on aging model *in vivo* are needed.

## 5. Conclusion

The present study reveals for the first time that *Ganoderma lucidum*-derived triterpenoid GA-D may exhibit potent antisenescence effects against H_2_O_2_-induced premature senescence of hAMSCs through the activation of PERK/NRF2 signals. These findings not only provide a deeper understanding of the antiaging effects of *Ganoderma* triterpenoid compounds but also provide a new theoretical basis for the development of antiaging supplements and the prevention of aging-related diseases on the basis of stem cell theory of aging.

## Figures and Tables

**Table 1:** Primer sequence of target genes.

Gene	Sequence (5′→3′)	GenBank ID	Length of product (bp)
*14-3-3ζ*	For: CCTGCATGAAGTCTGTAACTGAG Rev: GACCTACGGGCTCCTACAACA	NM_001135702.1	100
*PRDX3*	For: ACAGCCGTTGTCAATGGAGAG Rev: ACGTCGTGAAATTCGTTAGCTT	NM_006793.4	152
*SIRT1*	For: TAGCCTTGTCAGATAAGGAAGGA Rev: ACAGCTTCACAGTCAACTTTGT	NM_001142498.1	160
*SIRT2*	For: CACGCAGAACATAGATACCCTG Rev: CAGTGTGATGTGTAGAAGGTGC	NM_001193286.1	162
*SIRT3*	For: GACATTCGGGCTGACGTGAT Rev: ACCACATGCAGCAAGAACCTC	NM_001017524.2	122
*SIRT4*	For: GCTTTGCGTTGACTTTCAGGT Rev: CCAATGGAGGCTTTCGAGCA	NM_012240.2	79
*SIRT5*	For: GCCATAGCCGAGTGTGAGAC Rev: CAACTCCACAAGAGGTACATCG	NM_001193267.2	157
*SIRT6*	For: CCCACGGAGTCTGGACCAT Rev: CTCTGCCAGTTTGTCCCTG	NM_001193285.2	194
*SIRT7*	For: ACGCCAAATACTTGGTCGTCT Rev: AGCACTAACGCTTCTCCCTTT	NM_016538.2	119
*NRF2*	For: TGGGAGTAGTTGGCAGAT Rev: AGCGACGGAAAGAGTATGA	NM_006164.5	190
*β-Catenin*	For: GGGATGGTGGGTGTAAGA Rev: GCTGGTGACAGGGAAGAC	NM_001904.4	148
*ERK1*	For: CTACACGCAGTTGCAGTACAT Rev: CAGCAGGATCTGGATCTCCC	NM_002746.2	109
*ERK2*	For: TCTGGAGCAGTATTACGACCC Rev: CTGGCTGGAATCTAGCAGTCT	NM_138957.3	134
*β-Actin*	For: TGGCACCCAGCACAATGAA Rev: CTAAGTCATAGTCCGCCTAGAAGCA	NM_001101.3	186

## Data Availability

The data used to support the findings of this study are all presented in the Results section and are also available from the corresponding author (jhxiao@zmu.edu.cn, or jianhuixiao@126.com).

## Additional Points

*Highlights*. Ganoderic acid D retards the senescence of human amniotic mesenchymal stem cell. Ganoderic acid D exhibits potent protective effect against H_2_O_2_-induced premature senescence of hAMSCs without inducing cytotoxic side-effects. Ganoderic acid D retards hAMSC senescence through the activation of the PERK/NRF2 signaling pathway. Ganoderic acid D may act as a promising candidate for the antiaging agent discovery. Ganoderic acid D has been reported as the first antiaging tetracyclic triterpenoid compound.

## Supplementary Materials

Supplementary Materials Supplementary Figure 1: identification of hAMSCs.
