# Supplementary figures and images for: Ganoderic Acid D Protects Human Amniotic Mesenchymal Stem Cells against Oxidative Stress-Induced Senescence through the PERK/NRF2 Signaling Pathway
Source: Oxid Med Cell Longev. 2020 Jul 27;2020:8291413. doi: 10.1155/2020/8291413 (PMC7407022; doi:10.1155/2020/8291413)

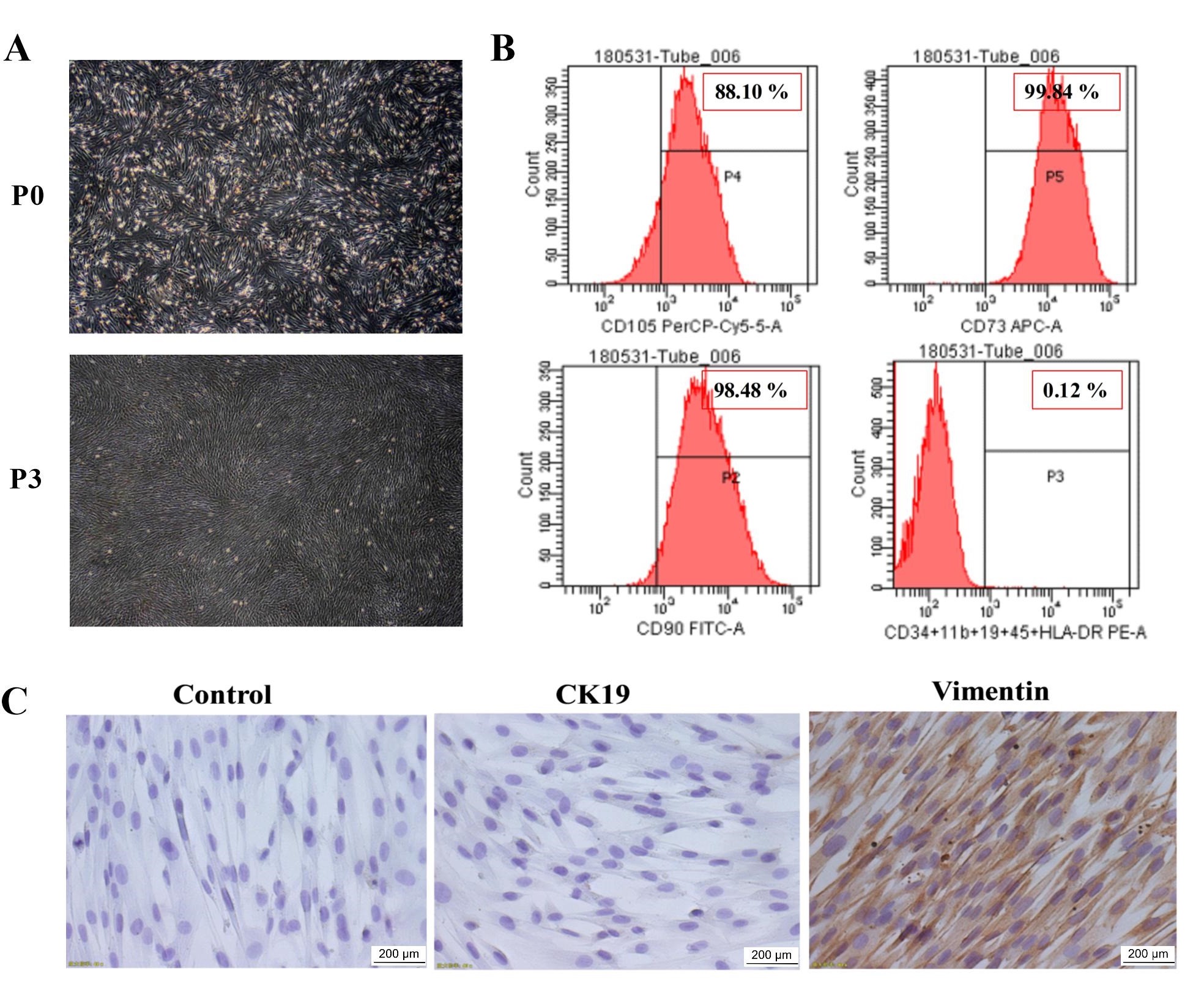

Supplement: Supplementary Materials — Supplementary Figure 1: identification of hAMSCs. [file 8291413.f1.zip › mat.8291413.v2.jpg]
